# Coronary Artery Bypass Graft Surgery for Spontaneous Coronary Artery Dissection in Early Pregnancy: Medical and Ethical Decision-Making Issues

**DOI:** 10.7759/cureus.35364

**Published:** 2023-02-23

**Authors:** Adrian G Yabut, Bradlee Bachar, Hussam Nagm

**Affiliations:** 1 Anesthesiology, Kaweah Health Medical Center, Visalia, USA; 2 Cardiothoracic Anesthesiology, Kaweah Health Medical Center, Visalia, USA

**Keywords:** coronary artery bypass grafting (cabg), angiography, acute coronary syndrome, myocardial infarction, pregnancy, spontaneous coronary artery dissection

## Abstract

Spontaneous coronary artery dissection (SCAD) in young females is a rare condition that typically occurs during the postpartum period.Even more rare is when SCAD occurs during the antepartum phase of pregnancy. This scenario can have catastrophic outcomes for both the mother and the unborn child. Due to its infrequency, there is minimal information on how to treat these patients effectively while keeping both the mother and the unborn child as safe as possible. We present a case of a 36-year-old multiparous Caucasian female brought in by an ambulance for sudden-onset left-sided chest pain radiating to her left shoulder, arm, and back. The initial laboratory tests were significant for a B-type natriuretic peptide (BNP) level of 190.82 pg/mL (the normal range is less than 100 pg/mL) and a troponin level of 3.98 ng/mL (the normal range is less than 0.04 ng/mL), which peaked to 18.39 ng/mL in less than 24 hours.

Electrocardiogram (EKG) showed sinus tachycardia of 103 beats per minute (bpm) and anterolateral ST-T changes suggestive of ischemia. Human chorionic gonadotropin (hCG) was 32 mIU/mL (the normal range is less than 5 mIU/mL), which may indicate early pregnancy. Echocardiogram (ECHO) showed left anterior descending (LAD) artery territory wall motion abnormalities, which included the akinesis of the apical, middle, and apical anterior septum and the hypokinesis of the basal anteroseptal segment. Her calculated ejection fraction was 38.4% with no valvular abnormalities. Cardiac catheterization showed severe diffuse LAD disease in the proximal segment with the middle LAD and diagonal branch subtotally occluded. The right coronary artery (RCA) had severe disease. Cardiothoracic surgery was consulted for a coronary artery bypass graft (CABG). The procedure performed was a three-vessel coronary artery bypass graft, which included the following: left internal mammary artery (LIMA) to middle LAD, saphenous vein graft (SVG) to distal LAD, and SVG to diagonal. This case report aims to provide additional information to the database of SCAD in pregnant females undergoing coronary artery bypass graft surgery.

## Introduction

Spontaneous coronary artery dissection (SCAD) in young females is a rare condition that typically occurs during the postpartum period. Even more rare is when SCAD occurs during the antepartum phase of pregnancy [1]. Havakuk and colleagues performed a retrospective cohort study evaluating 120 cases of SCAD; postpartum SCAD accounted for 73% of the time, of which 6% was during the second trimester, 18% during the third trimester, and 3% during the peripartum period (defined as 24 hours after or before delivery) [1]. Astonishingly, 0% of the cases were in the first trimester, making our case very rare. This scenario can have catastrophic outcomes for both the mother and the unborn child. Due to its infrequency, there is minimal information on how to treat these patients effectively while keeping both the mother and the unborn child as safe as possible. This case report aims to provide additional information and add to the database of SCAD in pregnant females undergoing coronary artery bypass graft (CABG) surgery.

Spontaneous coronary artery dissection (SCAD) is defined as a separation of the coronary artery wall creating a false lumen. Its etiology is non-atherosclerotic, nontraumatic, and not iatrogenic. This can lead to myocardial ischemia or infarction if the false lumen impinges into the true lumen [2]. The most commonly affected vessels are the left main or left anterior descending (LAD) artery [3]. Risk factors include pregnancy, multiparity, connective tissue disease, and emotional and physical stress [4]. It also typically affects females more than males, with the mean ages between 44 and 46 years old [2]. Patient symptoms typically present with chest pain, nausea and/or vomiting, dyspnea, and diaphoresis [5]. Complications of SCAD can lead to ventricular fibrillation, ST-segment elevation myocardial infarction (STEMI), and even sudden death [6]. SCAD is diagnosed primarily with the use of invasive coronary angiography, but it can be aided with the use of intravascular ultrasound, computed tomography angiography, and optical coherence tomography [6]. Treatment options for SCAD include conservative management with medical therapy, coronary artery stenting, or coronary artery bypass graft, which all depend on the extent and location of the injury(s). In this case report, we describe a multiparous 36-year-old female who presented to the emergency room for chest pain and was subsequently diagnosed with spontaneous coronary artery dissection.

## Case presentation

A 36-year-old multiparous Caucasian female with a past medical history of hypertension was brought in by ambulance for sudden-onset left-sided chest pain that radiates to her left shoulder, arm, and back while showering. Her history of hypertension developed after her last pregnancy in 2011, and she admitted that she has not been compliant with her medication. She has had five pregnancies and has four children. The patient denied any social history of smoking, drinking, or illicit drug use.

She stated that her pain started one hour prior to arrival and rated the pain 8/10. She endorsed nausea and vomiting but denied any shortness of breath or diaphoresis. She mentioned that she had similar symptoms two weeks prior at a local fair and was seen by the medic on site. Her systolic blood pressure at that time was elevated to approximately 180 mmHg, which resolved without medication after 15-20 minutes of rest.

**Figure 1:**
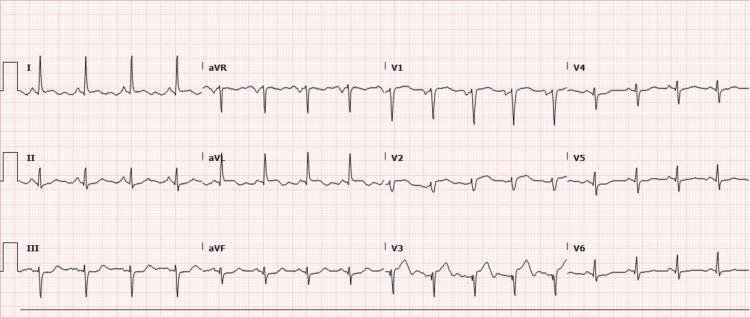
EKG showing sinus tachycardia of 103 bpm and anterolateral ST-T changes EKG, electrocardiogram; bpm, beats per minute; aVR, augmented vector right; aVL, augmented vector left, aVF, augmented vector foot

The initial laboratory tests were significant for a B-type natriuretic peptide (BNP) level of 190.82 pg/mL (the normal range is less than 100 pg/mL) and a troponin level of 3.98 ng/mL (the normal range is less than 0.04 ng/mL), which peaked to 18.39 ng/mL in less than 24 hours. Electrocardiogram (EKG) showed sinus tachycardia of 103 beats per minute (bpm) and anterolateral ST-T changes suggestive of ischemia, as shown in Figure 1. Human chorionic gonadotropin (hCG) was 32 mIU/mL (the normal range is less than 5 mIU/mL), which may indicate early pregnancy.

**Figure 2:**
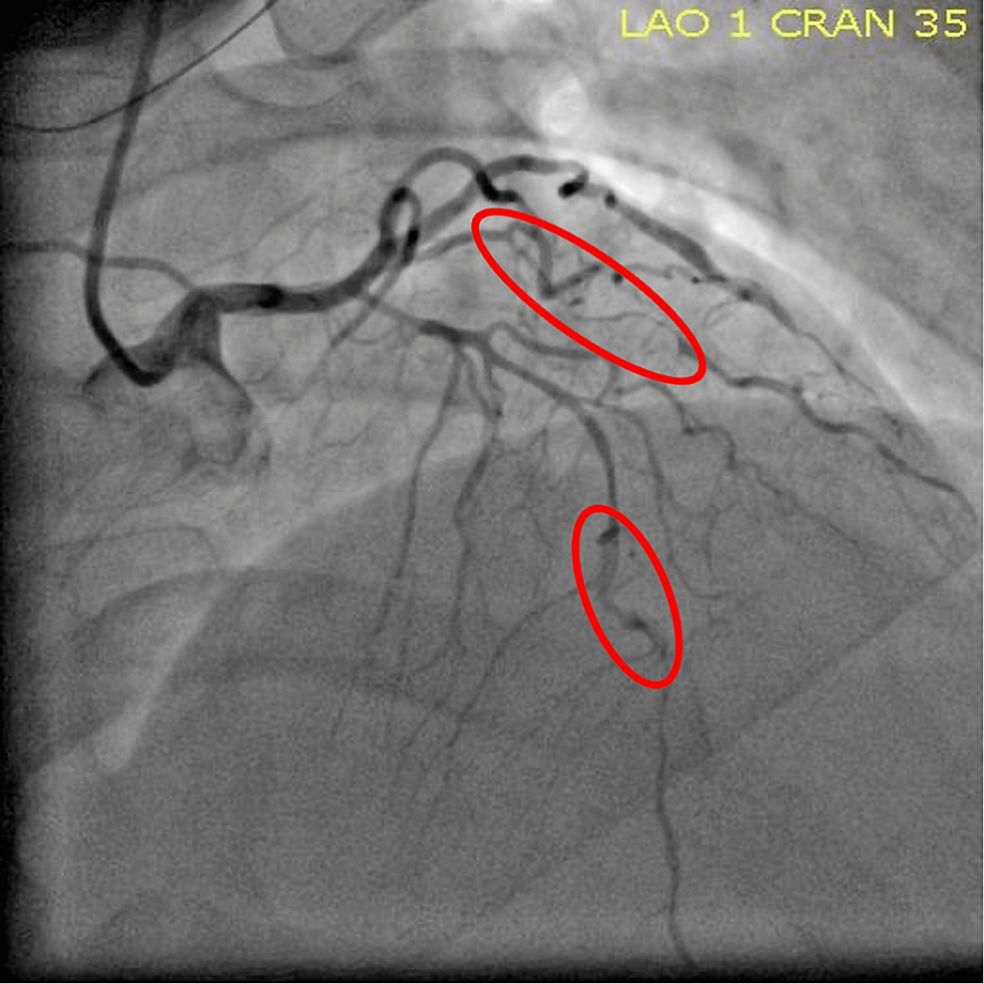
Flow-limiting dissection in LAD and diagonal coronary artery branches LAD: left anterior descending

**Figure 3:**
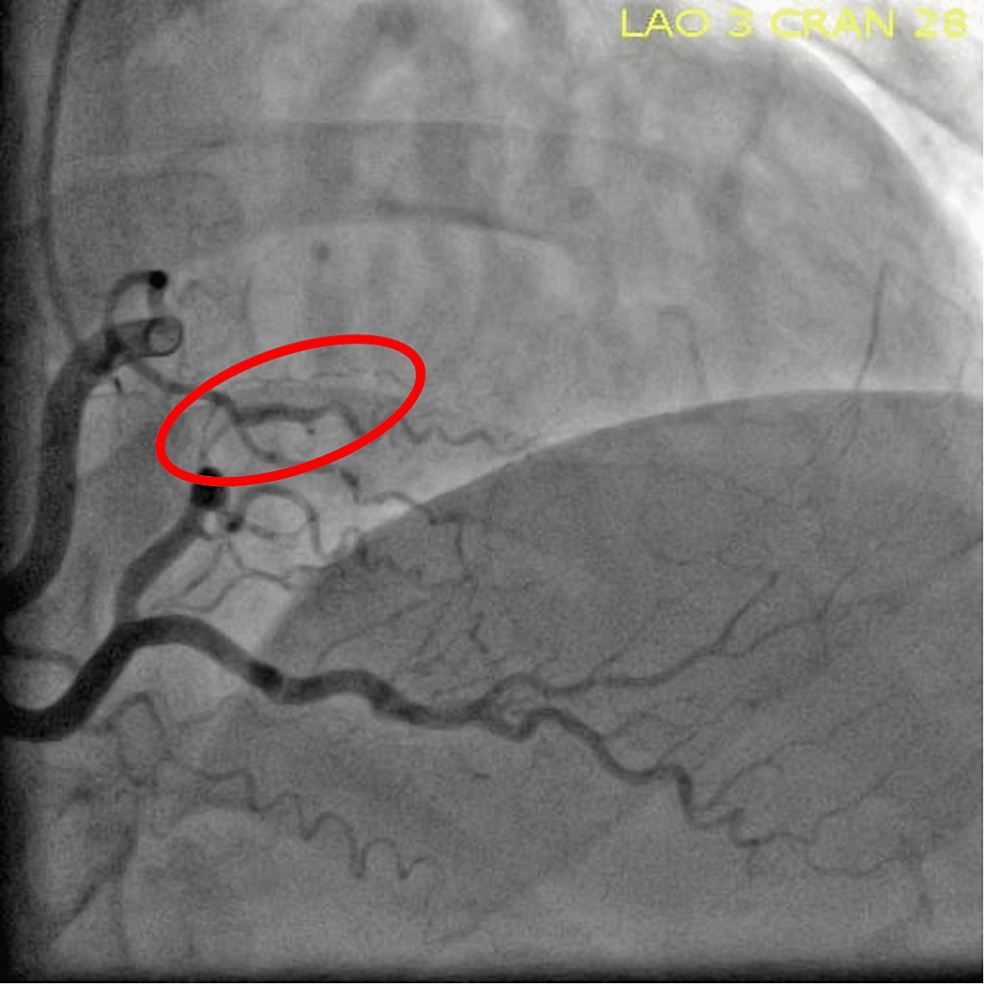
PLA branch of RCA with dissection PLA, posterolateral artery; RCA, right coronary artery

Cardiology was consulted for non-ST-segment elevation myocardial infarction (NSTEMI). Recommendations included an echocardiogram (ECHO), starting enoxaparin sodium, nitroglycerin, and a cardiac catheterization. Echocardiogram (ECHO) showed left anterior descending (LAD) artery territory wall motion abnormalities. Her calculated ejection fraction was 38.4% with no valvular abnormalities. Cardiac catheterization showed severe diffuse LAD disease in the proximal segment with the middle LAD and diagonal branch subtotally occluded, as shown in Figure 2. The posterolateral artery (PLA) branch of the right coronary artery (RCA) had severe disease, which is shown in Figure 3.

Obstetrics-gynecology was consulted and recommended a repeat hCG in the next 48 hours and to obtain a pelvic ultrasound to ensure no evidence of intrauterine or ectopic pregnancy. The repeat hCG level was 58 mIU/mL (the normal range is less than 5 mIU/mL), and her last menstrual period was 17 days ago, and she stated that she has been attempting to get pregnant. Ultrasound of the pelvis showed no signs of intrauterine pregnancy or ectopic pregnancy.

Cardiothoracic surgery was consulted for a coronary artery bypass graft (CABG). This recommendation was complicated given her elevated hCG and pregnancy. By proceeding with surgery, fetal demise is highly likely given the length of the procedure and the use of cardiopulmonary bypass (CPB) machine and anesthesia. Even if the fetus survives, there is no way to determine if the fetus would suffer any abnormalities. After a thorough discussion of the risks and benefits of the procedure, she decided to proceed. The patient signed the consent understanding all the risks associated with the surgery and her pregnancy.

Transesophageal echocardiogram prior to cardiopulmonary bypass showed the hypokinesis of the inferior, lateral, and anterior ventricular walls, as shown in Videos 1-2. Bypass time was 171 minutes, and cross-clamp time was 141 minutes. Upon visualization during surgery, the LAD had a spiral dissection involving the entire length of the LAD, first diagonal and second diagonal. The procedure performed was a three-vessel coronary artery bypass graft, which included the following: left internal mammary artery (LIMA) to middle LAD, saphenous vein graft (SVG) to distal LAD, and SVG to diagonal. Upon weaning from cardiopulmonary bypass, the ventricular function was not optimal despite norepinephrine and epinephrine, so a decision was made to place an intra-aortic balloon pump. Once placed, the cardiac contractility improved enough to come off bypass with the help of norepinephrine and epinephrine for cardiac and pressure support. The patient was then brought to the cardiovascular intensive care unit, intubated, and subsequently extubated one day later. Epinephrine, norepinephrine, and the intra-aortic balloon pump were discontinued after two days. Her hCG continued to increase and was 402 mIU/mL (the normal range is less than 5 mIU/mL) on day 3 postoperatively. A repeat ECHO five days postoperatively showed an ejection fraction of 35%-40%, a moderate left ventricular systolic dysfunction, and an improvement of the hypokinesia of the entire anterior septum, middle and apical anterior wall, and the apex. The patient was then discharged home on the fifth day.

**Video 1:** Transesophageal echocardiogram: transgastric mid-papillary short-axis view The hypokinesis of the inferior, lateral, and anterior left ventricular walls

**Video 2:** Transesophageal echocardiogram: midesophageal four-chamber view The hypokinesis of the inferior, lateral, and anterior left ventricular walls

## Discussion

The presumed pathophysiology of SCAD is due to hormonal changes in pregnancy; however, it is still not fully understood. Estrogen and progesterone receptors are thought to be key players as they are present in coronary arteries. Pregnancy has an increase in these hormones, which causes the weakening of connective tissue and collagen leading to arterial wall rupture and/or intramural hematoma [7]. Multiparous females have an accumulation of these hormones, which may explain their higher risk of SCAD. Hemodynamic changes during pregnancy such as increased cardiac output and increased intravascular volume may contribute to the rupture of a weakened vessel wall [3].

The majority of patients with SCAD (70%-97%) spontaneously heal, and conservative management is the preferred treatment. Patients with SCAD are recommended to start on aspirin and beta-blockade [4]. Low-dose aspirin is safe in both pregnant and breastfeeding mothers. The beta-blocker of choice is labetalol as the other medications may cause fetal growth restriction and bradycardia in feeding mothers. A 2017 multivariable analysis report from Vancouver included 327 patients and demonstrated a hazard ratio of 0.36 for the recurrence of SCAD, which reinforces its recommended use. Clopidogrel use should be individualized for each patient as there is no clear data on its safety during pregnancy. For breastfeeding, however, clopidogrel is not recommended. Treatment options include percutaneous coronary intervention (PCI) or CABG surgery. In Havakuk and colleagues’ retrospective cohort study evaluating 120 cases of SCAD, CABG surgery was performed in four pregnant females; in one case, the baby was born prematurely at 35 weeks (four weeks after the surgery), one case required immediate cesarean section due to fetal distress, and the remaining two cases resulted in fetal loss [1]. Since SCAD occurs mostly during the postpartum period, recommendations and information on how to manage a pregnant patient during a CABG are very limited. Unfortunately, after all the treatment the patient has endured, future pregnancies may exacerbate the recurrence of SCAD with reports as high as 30% [8]. Knowing this creates an ethical dilemma if the patient is trying to conceive. A survey report with mainly cardiologists showed that 58% discouraged pregnancy after having a SCAD [9].

The case above describes a multiparous 36-year-old female experiencing chest pain. Her chest pain symptoms began two weeks earlier, and she did not seek further medical evaluation. Her echocardiogram showed multiple wall motion abnormalities. PCI was not an option as the true coronary lumen could not be identified. Attempting to perform a PCI in this scenario could worsen the dissection or result in an emergency CABG [1]. For these reasons, undergoing a CABG was her only opportunity for revascularization. A team composed of an obstetrician, a cardiologist, a cardiothoracic surgeon, a neonatologist, a cardiac anesthesiologist, and the patient must determine the risks and benefits of cardiac surgery with cardiopulmonary bypass in a pregnant female. Cardiopulmonary bypass (CPB) has a maternal mortality ranging from 1.5% to 5% where fetal mortality is between 16% and 33% [10]. Uteroplacental perfusion and ultimately fetal development are affected during CPB. Adverse effects include alteration in coagulation and blood components, complement activation, vasoactive substance release, nonpulsatile flow, hypothermia, air and particular embolism, and hypotension [11]. This case now developed into a difficult ethical situation: the patient’s need for a CABG and her current pregnancy.

On postoperative day 3, her hCG continued to increase to 402, likely showing fetal growth. The cardiologist, cardiothoracic surgeon, and obstetrician all recommended to terminate her pregnancy as soon as possible. Continuing with pregnancy will increase the stress on her heart from increased cardiac output and intravascular volume, thus increasing her morbidity and mortality by further exposing her coronary vessel to pregnancy hormones. The abnormalities of the fetus at this stage are still unknown. The fetus has experienced anesthesia and cardiopulmonary bypass for hours and is too early to test for any abnormalities or viability. During her last visit with her obstetrician at six weeks and two days, intrauterine pregnancy was identified along with a heartbeat. The patient already has four children and was planning to have another. After a thorough discussion with her family, a decision was made to terminate her pregnancy for her well-being after hearing all the physician recommendations. The choice to terminate her pregnancy might have been a little easier since she has four children already. However, if this was her first child after years of trying to conceive, the decision might have been different. The termination of an infant using oral medications requires that the pregnancy be less than 10 weeks. The patient was six weeks and six days pregnant upon fetal termination. This decision can be very difficult, especially in couples trying to conceive.

A high clinical suspicion of SCAD in pregnant patients who present with chest pain is critical. Pregnant patients are usually of younger age and have no cardiovascular past medical history. Unfortunately, this may cause clinicians to dismiss true acute coronary syndrome. It can be easily unrecognized and have catastrophic outcomes if not diagnosed promptly.

## Conclusions

This case focuses on the dilemmas between medical and ethical issues of SCAD in early pregnancy. The SCAD case presented involved a 36-year-old female who was 17 days pregnant and underwent a CABG. This case is extremely rare as it is one of the first documented SCAD that occurred in the first trimester and one of five cases that underwent CABG. With the uncommon nature of SCAD in pregnancy, guidelines and recommendations are scarce. This case report hopes to add value to the medical community by providing medical management and an ethical scenario that other providers might encounter.
